# *Fusarium verticillioides* pigment: production, response surface optimization, gamma irradiation and encapsulation studies

**DOI:** 10.1186/s12896-024-00909-7

**Published:** 2024-10-30

**Authors:** Mai Ali Mwaheb, Yasmeen A. Hasanien, Amira G. Zaki, Alaa S. Abdel-Razek, Laila R. Abd Al Halim

**Affiliations:** 1 Botany Department, Faculty of Science, Fayoum University, Fayoum, 63514 Egypt; 2 Plant Research Department, Nuclear Research Center, Egyptian Atomic Energy Authority, Cairo, Egypt; 3 Radiation Protection and Safety Department, Hot Labs Center, Egyptian Atomic Energy Authority, Cairo, Egypt; 4 Agricultural Microbiology Department, Faculty of Agriculture, Fayoum University, Fayoum, 63514 Egypt

**Keywords:** *Fusarium verticillioides*, Natural pigment, Statistical optimization, Gamma irradiation, Encapsulation

## Abstract

**Background:**

Natural pigments are becoming more significant because of the rising cost of raw materials, pollution, and the complexity of synthetic pigments. Compared to synthetic pigments, natural pigments exhibit antimicrobial properties and is less allergic. Pigments from microbial sources could easily be obtained in an inexpensive culture media, produced in high yields, and microbes are capable of producing different colored pigments. Searching for new sources for natural pigments to replace synthetic ones in food applications has become an urgent necessity, but the instability of these compounds is sometimes considered one of the obstacles that reduce their application. Encapsulation provides an ideal solution for natural dye protection through a controlled release strategy. Thus, this study aims at isolation of several soil fungi and subsequent screening their pigment production ability. The chosen pigment-producing fungal strain underwent full identification. The produced pigment was extracted with ethyl acetate and estimated spectrophotometrically. As there is a necessity to obtain a high pigment yield for efficient industrial application, the best production medium was tested, optimum conditions for maximum dye production were also investigated through the response surface methodology, and gamma irradiation was also employed to enhance the fungal productivity. Encapsulation of the produced pigment into chitosan microsphere was tested. The pigment release under different pH conditions was also investigated.

**Results:**

A new strain, *Fusarium verticillioides* AUMC 15934 was chosen and identified for a violet pigment production process. Out of four different media studied, the tested strain grew well on potato dextrose broth medium. Optimum conditions are initial medium pH 8, 25 °C-incubation temperature, and for 15-day incubation period under shaking state. Moreover, a 400 Gy irradiation dose enhanced the pigment production. Chitosan microsphere loaded by the pigment was successfully prepared and characterized by infrared spectroscopy and scanning electron microscopy.

**Conclusion:**

This irradiated *Fusarium* strain provides a more economically favorable source for production of a natural violet dye with an optimum productivity, enhanced yield, and improved properties (such as, enhanced stability, controlled release, and bioaccessibility) by encapsulation with chitosan for efficient application in food industry.

## Introduction

Color is believed to be the primary sensory attribute that plays a vital role in the acceptance of foods to improve their actual appearance and quality [1]. In recent years, there has been a notable increase in the overuse of synthetic pigments, posing a threat to human health and the environment. In addition to being possibly carcinogenic, teratogenic, and non-biodegradable, synthetic colors can change the flavor of food. Due to their toxicity, several countries have outlawed the use of certain dyes, including Blue FCF, Blue No.1, and Blue No. 2 [2]. Also, customers are worried about synthetic food coloring's safety for their health [3]. As a result, alternative channels must be established for the production of pigments, such as finding natural sources for pigments manufacturing [4].

There are many different sources of natural pigments, including bacteria, fungi, algae, edible plants, seeds, and roots. Microbial pigments exhibit numerous benefits in manufacturing processes, including their ability to withstand seasonal fluctuations unlike plants, ease of production at large volumes, low maintenance costs, and structural stability [5]. Furthermore, the majority of filamentous fungi generate a range of pigment colors, including orange, bronze, yellow, red, Violet, and brown [6]. Fungi produce these colors as secondary metabolites in response to nutrient shortages [7].

Natural pigments offer numerous health advantages and have a high level of antioxidant activity. The human body's ability to effectively utilize these bioactive compounds is determined by their bioaccessibility and bioavailability [1].

Because natural pigments are poorly bioavailable and chemically unstable, incorporating them into food products is extremely difficult. An excellent approach to improve its digestibility, controlled release, and bioaccessibility is through encapsulation. The term "microencapsulation" refers to a technology that packs liquid, solids, or gaseous substances into tightly sealed capsules with controlled release rates under particular circumstances [8]**.** Microcapsules are now widely used in a variety of industries, such as pharmacy, food processing, agriculture, and medicine. Effective encapsulation technologies are necessary during food manufacturing in order to preserve pigment bioavailability in the human gastrointestinal tract and prevent pigment degradation [1].

N-acetyl-D-glucosamine and β-(1–4)-linked D-glucosamine make up the linear polymer of chitosan. It is created through the process of alkaline deacetylation of chitin, the primary building block of crustaceans like shrimp, crabs, and crawfish. After cellulose, chitin is the second most common natural polymer[9]. The degree of de-acetylation affects chitosan's physicochemical properties, as well as its biodegradability and immunological activity. Moreover, chitosan's biocompatibility, biodegradability, low toxicity, and biological activities, which include lowering cholesterol levels and demonstrating antimicrobial qualities, make it beneficial in food industries [10].

The pigment productivity by fungi can be boosted by selecting the suitable strain, carrying out the proper fermentation process, and choosing the appropriate media [11]. Also, gamma radiation has strong effects on enhancing the microbial productivity as it classified among the ionizing radiation. It induces alterations in the genes of cells through the DNA repair mechanisms within the cells [12]. The development of cost-effective and viable technologies for the preparation of natural food color is the major aim of this study. Hence, this research paper was prepared in order to search for a new pigment producing fungal strain. In addition, the potential for growth and pigment production from the pigment producing strain was assessed by optimizing the culture medium, initial pH, incubation temperature, incubation period, and incubation state were optimized. Boosting the pigment yield by the producing fungal strain through a gamma irradiation employment was also investigated. Moreover, preparation and characterization of chitosan beads containing the produced pigment were also explained for a prospective application in the food industry.

## Material and methods

### Isolation of pigment-producing fungi

Several fungal isolates were recovered from the farm soil of Faculty of Science, Fayoum University, Fayoum, Egypt. The collected soil samples were placed in clean and sterile plastic bags and were directly transferred to the laboratory for isolation and purification of pigment producing fungi. Soil samples were suspended in a sterile distilled water, shaked at room temperature for 15 min., serially diluted, and 100 µl were surface-inoculated on a potato dextrose agar (PDA; Bacto, Sparks, MD, USA) containing plates with the following composition: potato (200 g.l^−1^), glucose (20 g.l^1^) and agar (20 g.l^−1^). Plates were incubated at 25 °C for 7 days. After cultivation, fungal isolates which showed reverse pigmentation were purified and preserved at refrigerator on PDA slants to be ready for the next pigment extraction experiment.

### Morphological identification of the pigment-producing fungus

The primary method of examining the macroscopic appearance of the chosen fungal isolate was to cultivate it on a potato dextrose agar (PDA) medium for seven days at 25 °C. Additionally, the slide culturing methodology was employed at the Assuit University Myological Center's (AUMC) for determining the strains' microscopic morphology.

### Molecular identification

Patho-gene-spin DNA/RNA extraction kit (Intron Biotechnology Company, Korea) was used at the Molecular Biology Research Unit, Assiut University, to perform the DNA extraction protochol. SolGent Company (Daejeon, South Korea) provided assistance with sequencing and polymerase chain reaction (PCR) techniques. By utilizing the universal primers ITS1 (forward) and ITS4 (reverse), which were added to the reaction mixture, the ITS sections in the rRNA gene of the selected fungus were amplified. ITS1 (5'-TCCGTAGGTGAA CCTGCGG—3') and ITS4 (5'-TCCTCCGCTTATTGATATGC -3') are the two-primer compositions. After adding ddNTPs to the reaction mixture, the purified PCR products were sequenced using the same primers**.** The National Center of Biotechnology Information (NCBI) website's Basic Local Alignment Search Tool (BLAST) was used to examine the acquired sequences. By employing the MegAlign (DNA Star) software version 5.05., a phylogenetic tree was established.

### Pigment production

For pigment production by the identified fungal strain, 1 ml of a prepared spore suspension (1*10^8^ spores.ml^−1^) was added to 50 ml of the seed culture medium (potato dextrose broth, PDB). The seed culture was grown at room temperature on a rotary shaker adjusted at 25 °C ± 2.0 °C, 120 rpm for 7 days in a 250 ml flask with of potato dextrose broth medium. As a negative control, medium without a fungal inoculation was employed.

### Pigment yield estimation

For pigment quantification, a clear and impurity-free pigment supernatant was primarily prepared by placing samples of the fermented culture broth containing the produced pigment in 50 ml Falcon tubes and were centrifuged at 10,000 rpm for 20 min. Subsequently, the pigment was extracted using multiple solvents in accordance with the reported pigment extraction methodology [13]. The most optimal solvent for achieving the highest possible pigment extraction was chosen (data not shown). Using a spectrophotometer (JASCO Japan model V-560), pigment absorbance was determined following ethyl acetate extraction. To find the optimal wavelength for maximum light absorption—that is, the wavelength that gives the most significant absorbance of the pigment's violet color—the pigment was scanned at various wavelengths (between 200 and 700 nm). The fungal-free medium was used as a blank. As a result, the pigment yield in this study was described as the extract's optical density at 380 nm (OD 380).

### Dry biomass yield

After fungal fermentation, filtration was applied to obtain the mycelial wet biomass yield. The yielded biomass was then washed by distilled water and dried in an oven at 50°C until its weight remained constant [14]**.**

### Pigment production optimization approaches

First, the most favored medium for pigment production by the tested fungal strain was elected using the traditional method of one-factor-at-a-time (OFAT) protocol. The selection of the factors and their ranges to be studied was based on their significant effect on pigment productivity process, according to previous studies [15, 16]. The optimal values for the most important variables in the pigment production process were subsequently determined using a Box-Behnken Design (BBD). Accordingly, the optimization methodology was established as follows:

### Screening the production media through the OFAT approach

Four cultivation media, Czapek-Dox's broth (CZB) [17]; yeast dextrose broth (YDB) [18]; Potato dextrose broth (PDB) [19]; and malt extract broth (MEB) [20] were screened in this experiment to select the best one for maximum pigment production by the selected fungal strain. Accordingly, mycelial plugs of approximately 6 mm in diameter was cultivated in 500 ml Erlenmeyer flasks contained 200 ml of the tested medium, each medium was tested separately. Incubation of the prepared broth cultures was carried out for 7days in a shaking incubator adjusted at 150 rpm and 25 °C. The pigment yield at each tested medium was evaluated and the best pigment production medium was employed in the next response surface optimization experiment. For each set of conditions, duplicate experiments were conducted. After fermentation, the amount of dry biomass and produced pigment was quantified.

### Response surface optimization approach

Four process parameters as temperature, time, state of incubation, and initial medium pH were examined and response surface optimized in relation to their effects on the fungal pigment productivity. Each of the tested numerical factors was encoded at three different levels of analysis (+ 1 for the high level, 0 for the central level, and − 1 for the low level) as follows: pH (3–5-8), temperature (25–32.5–40 °C ± 2.0 °C), and fermentation time (5–10-15 days). While, the categorical factor of incubation state was encoded at only two levels of shake state and static state (Table 1).

**Table 1:** Studied variables and their levels in the applied Box-Behnken’s design

Variables		symbol	Coded and actual values			Unit
			-1	0	+ 1	
**Numerical variables**	**pH**	A	3	5.5	8	-
	**Temperature**	B	25	32.5	40	^o^ C
	**Time**	C	5	10	15	days
**Categorical variable**	**Incubation state**	D	(1) Static state	-	(2) Shaking state	-

Shaking Condition (120 rpm), Static Condition (0 rpm)

The tested process parameters and their levels were selected by searching the related reports [14, 21, 22]. Thirty pigment production experimental trials, including six central points, were conducted in the current BBD. The designed experimental runs in the employed BBD were duplicated and the mean absorbance was estimated and analysed. The maximum pigment absorbance was determined by means of analysis of variance, or ANOVA. The estimated impacts of the variables and the regression coefficients of the built models were computed. For the graphical analysis and regression, Minitab 18 Software (free trial version) was utilized.

### The effect of gamma irradiation on pigment yield

Separating hyper-producers could lower the overall cost of the production process when gamma irradiation mutagenesis is used to enhance microbial cultures [23]. The chosen gamma irradiation doses in this study were based on previous literature that showed that exposure of the fungal spores to low gamma irradiation doses enhances their bioactivity [24–26]. Accordingly, a prepared spore suspension (1*10^8^ spores.ml^−1^) was exposed to gamma irradiation at four radiation doses, 200, 400, 600, and 800 Gy, each was tested independently using a Cobalt-60 gamma cell (MC20, Russia), housed in the Nuclear Research Center of the Egyptian Atomic Energy Authority in Egypt, at a dose rate of 0.35 kGy.h^−1^. Accordingly, it should be noted that the irradiated spore suspensions were left overnight at 7ºC in darkness to prevent photoreactivation. The irradiated spore suspension was cultured in the obtained optimum culture medium under the optimum conditions obtained by the applied BBD and the pigment yield at each irradiation dose was measured.

### Encapsulation of the fungal pigment in chitosan beads

The chitosan beads containing the extracted pigment was prepared according to [9, 10, 27] with some modifications as follows:

1. chitosan dissolution by dissolving 2.0 g of chitosan in 98 ml distilled water and 2 ml of acetic acid (2%, v/v). The aqueous solution was strongly stirred over night at 60 °C.
2. 1 ml of the lyophilized pigment dissolved in methanol (0.1 gm.ml^−1^) were added to chitosan solution and mixed carefully. The solution was kept in refrigerator for 15 min to avoid bubbles’ formation.
3. Beads formation and chemical cross-linking: chitosan-pigment beads were formed through beads formation apparatus. Accordingly, 5 ml of the prepared solution was poured in syringe equipped with a micro-pipette tip.Using different tips, the droplet diameters were regulated. The solution was dropped via the syringe pump forcing, while still stirring, intoa 100 ml of the cross linking reagent of 0.5 N NaOH and shaken for 2 h at 50 °C. The pigment-carrying chitosan beads were separated through filtration, and then washed with deionized water and phosphate buffer (pH 7).
4. Beads activation by glutaraldehyde:5 gm beads were treated with 50 ml of 0.5% glutaraldehyde solution in phosphate buffer, pH 5.4. The activation process was conducted at room temperature for 3 h while being stirred at 150 rpm. Following this procedure, unreacted glutaraldehyde was eliminated from the activated beads by washing them with distilled water.

### Characterization of the chitosan-pigment beads

FT-IR spectra of dried pulverized samples of the free chitosan beads and pigment-loaded chitosan beads were applied with a spectrophotometer (VERTEX 80v, BRUKER, Germany) at 4 cm^−1^ resolution and measurement scale range of 4000–400 cm^−1^. The samples were prepared as KBr pellets.Moreover, to examine the morphology of the beads, samples were dried and subjected to scanning electron microscopy analysis (SEM, JEOL JSM-5600 LV, Japan).

### Release studies

The chitosan microspheres loaded with pigment underwent a release test. One gram of microspheres was put into fifty milliliter flasks and shaken at room temperature in ten milliliters of buffer solution with acidic pH values of 2.0, 3.0, 4.0, and 5.0. Three milliliter samples were taken out at pre-arranged intervals to measure the amount of pigment released, and they were then put back into the flask. The samples were examined with a UV–Vis spectrophotometer set to 380 nm. The calibration curve for the pigment under test was used to determine the percentage of pigment released. Every experiment was carried out in duplicate. The mean percentage of the pigment released was used to express the results.

### Statistical analysis

Minitab 18 Software (free trial version) was applied to design and analyze the response surface model. The statistical parameters were estimated using analysis of variance (ANOVA). An equation of second order polynomial was employed to establish a correlation between responses and independent factors. The following Equation (Eq. 1) illustrates the behavior of the system:

1 $$Y= {\alpha }_{0}+\sum\nolimits_{j=1}^{K}{a}_{j}{X}_{j}+\sum\nolimits_{j=1}^{K}{\alpha }_{jj}{X}_{j}^{2}+\sum_{j<1}{\sum }_{l=2}^{K}{\alpha }_{jl}{X}_{j}{X}_{l}+\varepsilon$$

where Xj and Xl are the independent factors, k is the number of independent parameters (k = 4 in this study), ε is the model error, and Y is the response (pigment yield,OD380). The intercept coefficient of the y axis is denoted by α0. The interaction coefficients of the linear and quadratic models are represented by αj, αjj, …, and αjl.

## Results

### Pigment producing fungal isolates

**Fig. 1:**
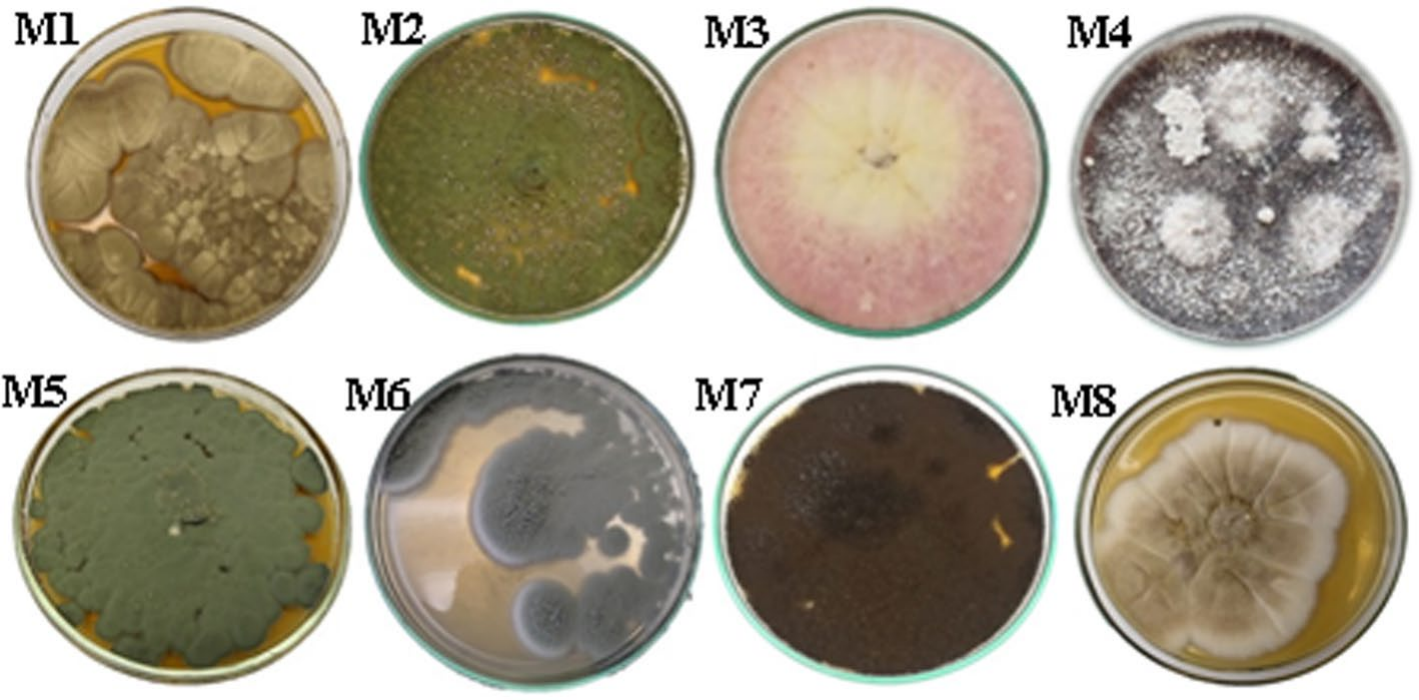
Pure cultures of the eight recovered pigment-producing fungal isolates grown on Potato dextrose agar plates after 7-day incubation

Out of many fungal isolates recovered from the soil samples, only eight isolates (encoded from M1 to M8) observed secretory pigments on the PDA medium. These isolates were morphologically belonged to *Penicillium*, *Fusarium*, *Alternaia*, and *Aspergillus* species (Fig. 1). This study focused on the M4 isolate that demonstrated a reverse violet pigmentation on PDA plates, and morphologically and molecularly detected as deposited in Assuit university mycological center for culture collection (AUMC) http://www.aun.edu.eg/aumc.htm.

### Fungal identification

**Fig. 2:**
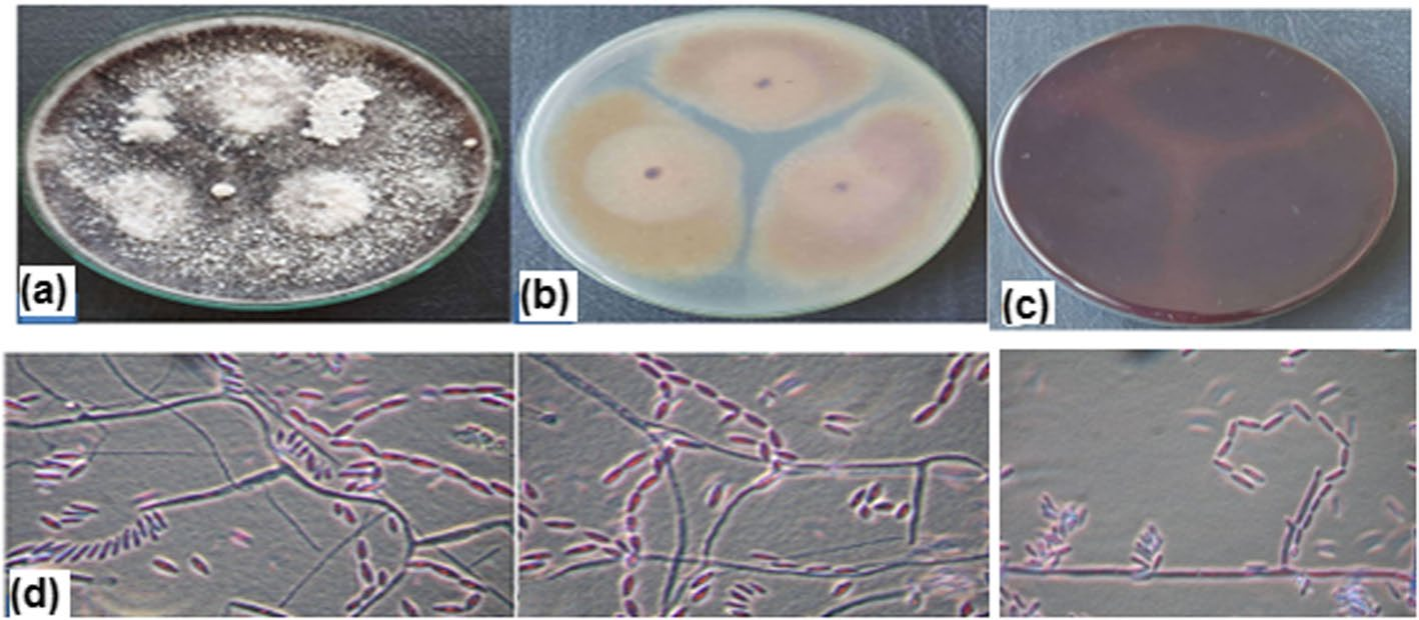
*Fusarium verticillioides* AUMC15935 morphology on potato dextrose agar after incubation at 25 °C. **a** shows the forward view while, **b** and **c** show the reverse view after 7 days and 15 days of incubation, respectively. **d** shows the microscopic features, the fungal hypha and the phialides bearing chains of microconidia

**Fig. 3:**
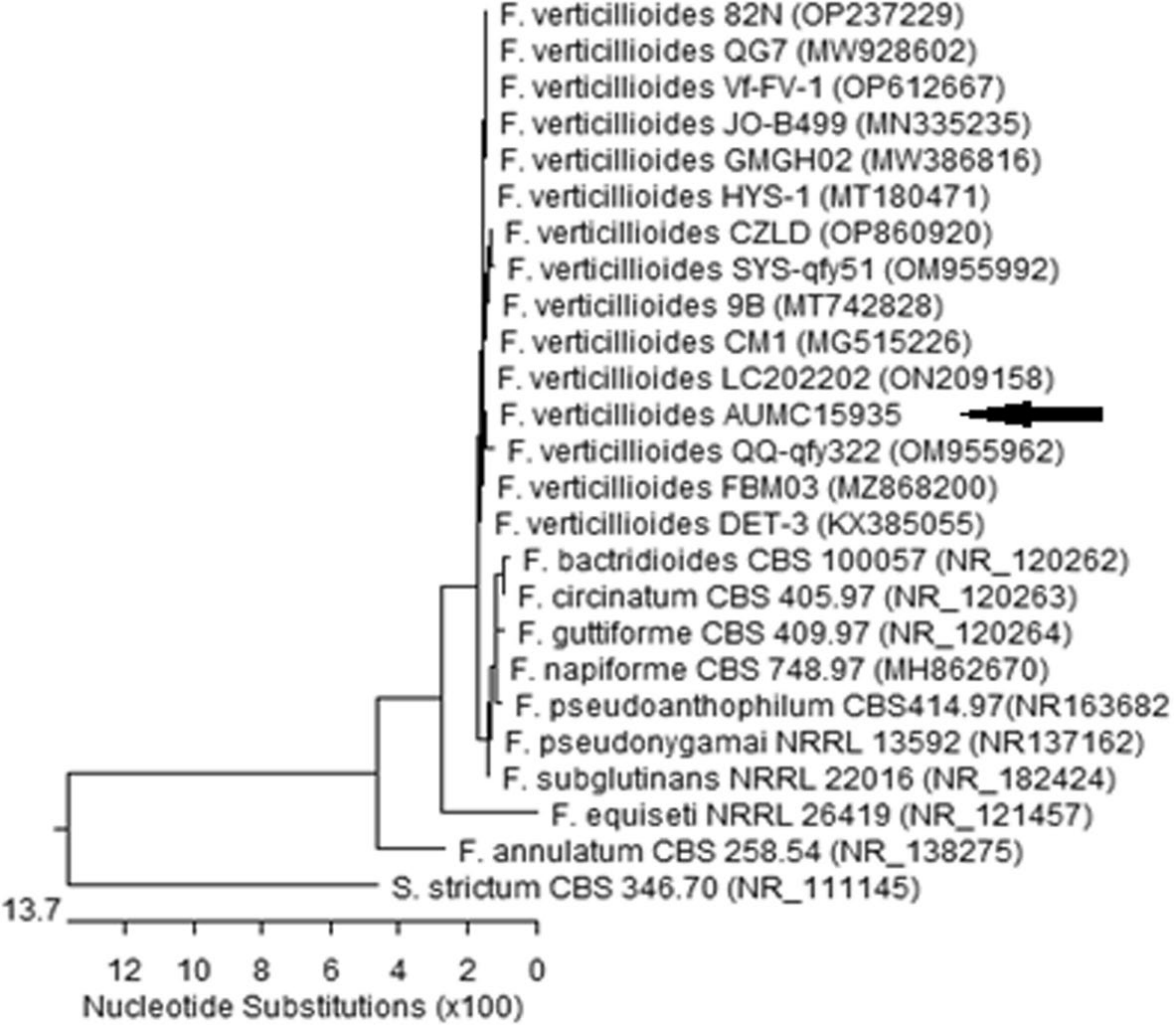
Phylogenetic tree based on ITS sequences of rDNA of the isolated *Fusarium verticillioides* AUMC15935 (arrowed) aligned with related sequences accessed from the GenBank. *Sarocladium strictum* is incluled as an outgroup strain. F. = *Fusarium, S. Sarocladium*

For morphological examination, the M4 isolate was cultured on potato dextrose agar (PDA) medium and incubated at 25°C ± 2.0 for 7 days [28]**.** The isolated strains grew rapidly on the potato dextrose agar (PDA) medium. As illustrated in Fig. 2, the forward examination shows white to pale salmon colonies with low and often ropy mycelium and a powdery texture as a result the formed chains of microconidia. The reverse examination shows a violet pigmentation. Via molecular studies, the sequenced 18 rDNA of the M4 strain showed 99.81–100% identity and 100% coverage with the *Fusarium verticillioides* strains. The constructed phylogenetic tree of the identified *Fusarium verticillioides* AUMC 15934 is illustrated in Fig. 3.

### Pigment production on different broth culture

**Fig. 4:**
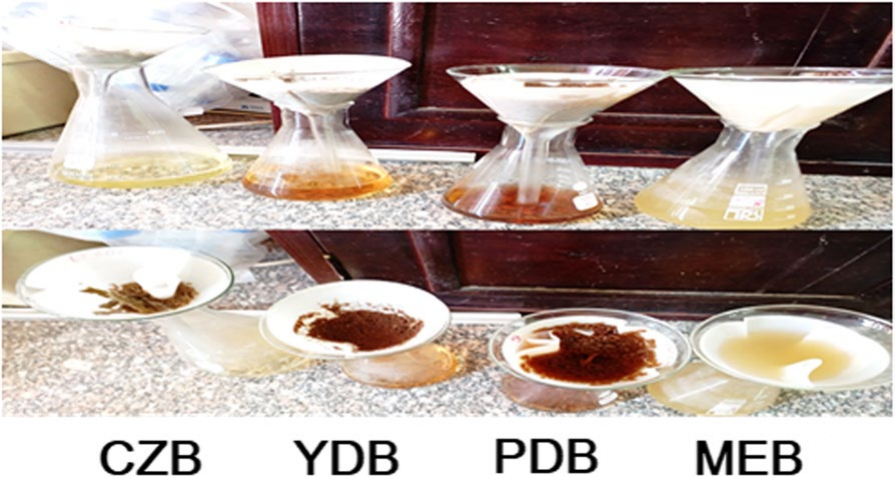
The harvested biomass and pigmentation of *Fusarium verticillioides* AUMC15935 after cultivation on various broth media

The growth medium significantly impacted the violet pigment production from *Fusarium verticillioides* AUMC 15934. Figure 4 shows the fungal pigmentation produced after cultivation on the four tested broth media. After scanning each medium filtrate via UV-scan spectrophotometer to determine the maximum absorption for the produced pigment at the best cultivation medium for efficient pigmentation (data not shown), the maximum absorption was obtained at 380 nm using PDB filtrate. Therefore, the pigment yield can be quantified indirectly by measuring its optical density at 380 nm (OD380) using a spectrophotometer. It was noted that the increase in the quantity of the cultured biomass was not necessarily accompanied by an increase in the yield of pigment produced as demonstrated in Fig. 4. As illustrated, *Fusarium verticillioides* could not give any pigmentation on CZB medium despite developing a moderate amount of biomass on the same medium.

### Statistical optimization for pigment production

#### The Box-Behnken design

**Fig. 5:**
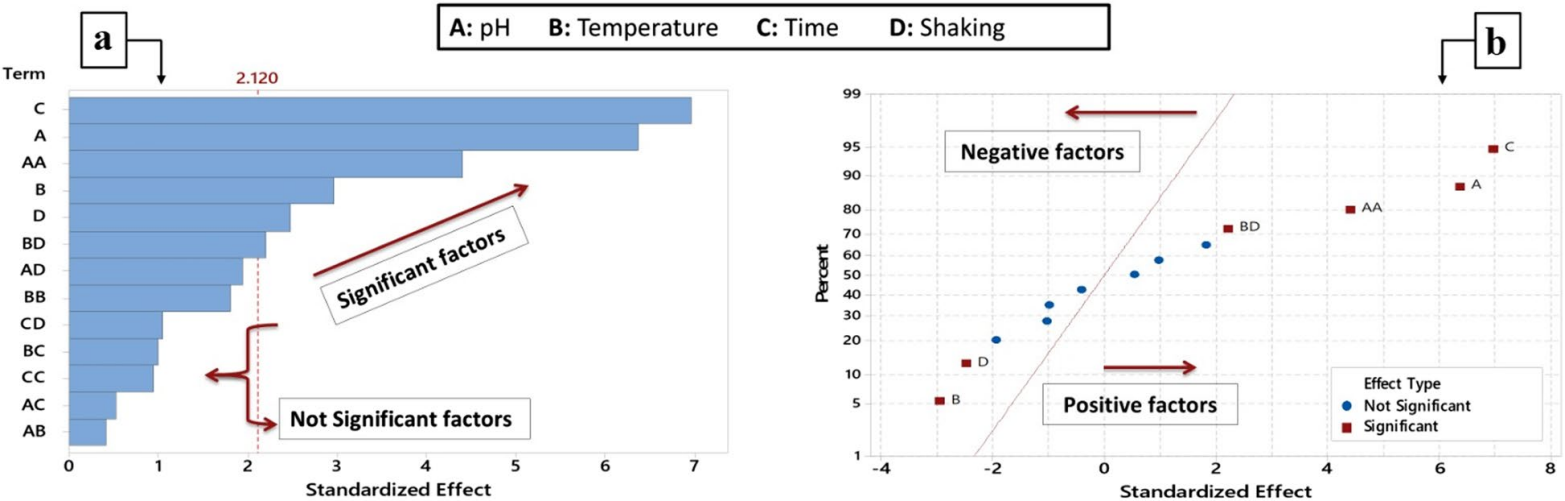
Graphical representation of BBD model results for pigment generation. The pareto chart (**a**) depicts the significant and non significant terms in the pigment production by *Fusarium verticillioides* AUMC15935. While, the normal plot (**b**) depicts the positive and negative terms affecting the pigment production process

The pigment yield (OD380) recovered at each of the thirty experimental model trials is displayed in BBD matrix in Table 2. The statistical analysis of the obtained data is shown in Table 3, and a pareto chart (Fig. 5a) provides a visual representation of the data. The ANOVA for the data from the BBD under examination showed that the model produced a significant difference (*P*-value < 0.05) in pigment production. Since the pigment yield varied from 0.035 OD380 (run 20) to 1.287 OD380 (run 17), highlighting the influence of the tested process conditions. Furthermore, positive T-values show a synergistic effect between the factors, whereas negative T-values show an antagonistic effect. Table 3 illustrates that the response surface regression analysis for the BBD was significant (model *P*-value < 0.05).

**Table 2:** The Box-Behnken design (BBD) matrix for four examined variables in coded values besides the observed and expected pigment yield exhibited in the model trials

**Run**	**Parameters**				**Dry weight** (g.l^−1^)	**Pigment yield (OD380)**	
	A	B	C	D		Observed	Predicted
1	-1	-1	0	1	4.81	0.400	0.286
2	+ 1	-1	0	1	1.18	0.507	0.611
3	-1	+ 1	0	1	1.25	0.117	0.275
4	+ 1	+ 1	0	1	1.13	0.523	0.524
5	-1	0	-1	1	1.72	0.092	0.072
6	+ 1	0	-1	1	0.85	0.359	0.311
7	-1	0	+ 1	1	6.13	0.441	0.408
8	+ 1	0	+ 1	1	1.65	0.807	0.743
9	0	-1	-1	1	1.52	0.062	0.047
10	0	+ 1	-1	1	0.85	0.073	0.089
11	0	-1	+ 1	1	1.51	0.407	0.522
12	0	+ 1	+ 1	1	0.39	0.466	0.381
13	0	0	0	1	1.51	0.132	0.128
14	0	0	0	1	1.35	0.135	0.128
15	0	0	0	1	1.25	0.129	0.128
16	-1	-1	0	2	3.16	0.408	0.420
17^a^	+ 1	-1	0	2	3.16	1.287	0.998
18	-1	+ 1	0	2	1.81	0.096	0.123
19	+ 1	+ 1	0	2	0.79	0.524	0.625
20	-1	0	-1	2	0.99	0.035	-0.004
21	+ 1	0	-1	2	0.72	0.413	0.487
22	-1	0	+ 1	2	2.8	0.459	0.467
23	+ 1	0	+ 1	2	6.27	0.932	1.055
24	0	-1	-1	2	1.92	0.063	0.239
25	0	+ 1	-1	2	3.12	0.138	-0.005
26	0	-1	+ 1	2	4.02	0.838	0.850
27	0	+ 1	+ 1	2	1.65	0.498	0.424
28	0	0	0	2	3.14	0.335	0.245
29	0	0	0	2	3.4	0.243	0.245
30	0	0	0	2	1.84	0.144	0.245

(−1) low level, (1) high level, (0) center point, A(initial pH), B(temperature ^o^C), C (fermentation time in days), D (shaking and static state), and ^a^ refers to the maximum pigment yield obtained under fermentation conditions at run 17

**Table 3:** Statistical analysis and regression statistics in the Box-Behnken design

Terms	Coef	*T* value	*F* value	*P* value
Model			10.54	0.000
Linear			25.99	0.000
A	0.2065	6.37	40.54	0.000
B	-0.0961	-2.96	8.78	0.009
C	0.2259	6.96	48.50	0.000
D	-0.0588	-2.48	6.15	0.025
Square			7.27	0.003
A*A	0.2103	4.40	19.39	0.000
B*B	0.0861	1.80	3.25	0.090
C*C	0.0457	0.96	0.92	0.353
2-Way Interaction			1.86	0.150
A*B	-0.0191	-0.42	0.17	0.683
A*C	0.0241	0.53	0.28	0.606
A*D	-0.0631	-1.95	3.79	0.069
B*C	-0.0458	-1.00	0.99	0.333
B*D	0.0714	2.20	4.85	0.043
C*D	-0.0340	-1.05	1.10	0.310
Error				
Lack-of-Fit			4.58	0.277
Model *R* ^*2*^	0.8954			
Model adjusted *R* ^*2*^	0.8104			

Coef. Coefficient, (−) T value indicates negative effect, (+) T value indicates positive effect

After investigating into the main, squared, and interaction impacts of the chosen factors, it was found that the following variables: A, B, C, D, A^2^, and BD significantly affected the production of pigment (P-value < 0.05). According to normal plot in Fig. 5b, the terms A, C, A^2^, and BD significantly increased the production of pigment. According to the obtained data of *P*-values of ANOVA table and its graphical explanation by the Pareto chart as well as data of T-values and its graphical representation by the normal plot, the pigment production process from *Fusarium verticillioides* affected significantly by all the tested factors as follows: an alkaline condition is more favoured than acidic one. The pigment yield increases by increasing the incubation period until reach the maximum after 15 days. For the interaction effect, the model showed a strong significant relation between the incubation temperature (Factor B) and the incubation state (Factor D). Accordingly, a higher pigment yield is achieved at incubation under low temperature with shaking state than that under high temperature with static state. A strong correlation and a well-fitting model were indicated by the model correlation coefficient (R^2^) of 0.8954 and the adjusted R^2^ of 0.8104, respectively. The applied BBD's lack-of-fit value was non-significant, meaning that the equation could predict pigment in all situations involving the variables under investigation. Our second-order polynomial equation effectively represents the true relationship between the response and experimental independent factors, according to the results of the ANOVA. The empirical relationship between the response and independent factors is represented by the equation. Since, pigment production in the PDB fermentation medium under static incubation conditions is covered by Eq. (2). In the meantime, pigment production under shaking conditions is covered by Eq. (3):

2 $$Pigment\;yield\left(OD380\right)at\;the\;static\;state=1.88-0.299\;\mathrm A-0.0850\;\mathrm B+0.0309\;\mathrm C+0.03364\;\mathrm A^2+0.001531\;\mathrm B^2+0.00183\;\mathrm C^2-0.00102\;\mathrm{AB}+0.00193\;\mathrm{AC}-0.00122\;\mathrm{BC}$$

3 $$pigment\;yield\left(OD380\right)at\;the\;shaking\;state=2.20-0.248\;\mathrm A-0.1041\;\mathrm B+0.0445\;\mathrm C+{0.03364\;\mathrm A}^2+{0.001531\;\mathrm B}^2+{0.00183\;\mathrm C}^2-0.00102\;\mathrm{AB}+0.00193\;\mathrm{AC}-0.00122\;\mathrm{BC}$$

**Fig. 6:**
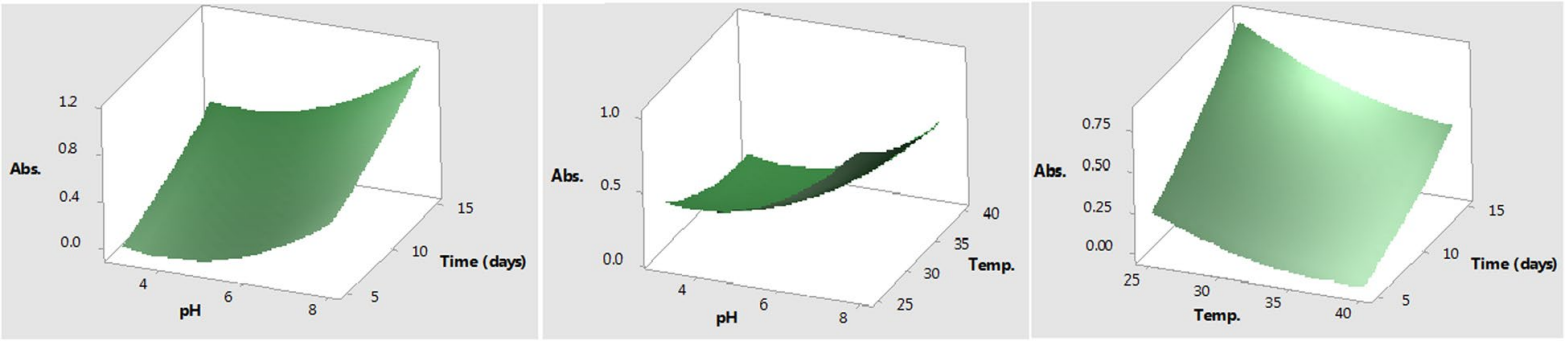
3D surface plots show the interaction effect of the tested factors on the pigment production by *Fusarium verticillioides* AUMC15935

Figure 6 shows surface plots that enable a visual examination of the interactive effects between each pair of parameters, with the third factor set to a middle value. According to the Minitab software v.18's response optimizer tool, the maximum pigment yield (1.3733) can be reached with 100% desirability at 25 °C of incubation temperature, pH 8 for the initial medium, and 15 days of incubation under shaking state. The verified mean pigment yield of 1.3220 at these identified optimal conditions was extremely near to the yield that was predicted.

#### Effect of gamma irradiation on pigment production

**Fig. 7:**
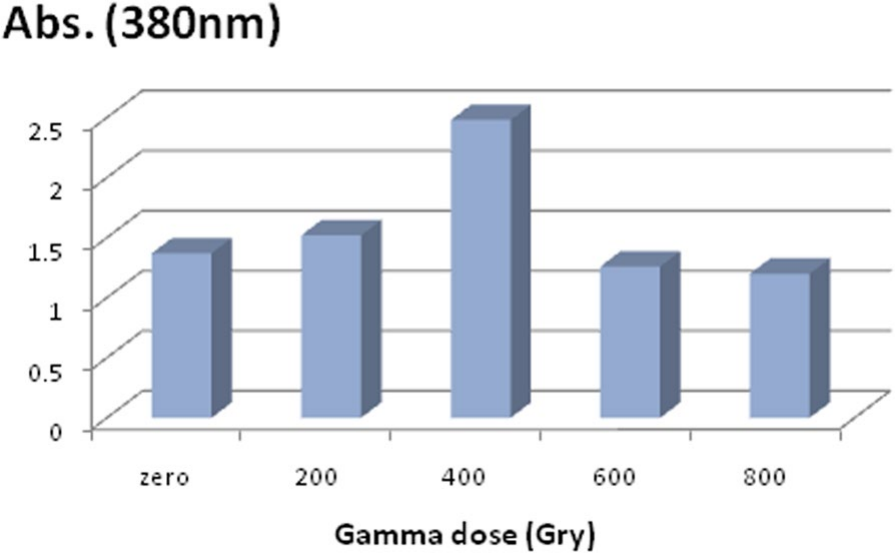
Enhancement of pigment production by gamma irradiation of *Fusarium verticillioides*AUMC15935

When comparing the pigment production by the irradiated tested fungal strain to that of the control (i.e., non irradiated), Fig. 7 demonstrated the impact of gamma irradiation. As can be observed, higher doses at 600 and 800 Gy led to a reduction in the pigment production. The dose that proved to be most effective was 400 Gy.

#### FTIR and SEM characterization

**Fig. 8:**
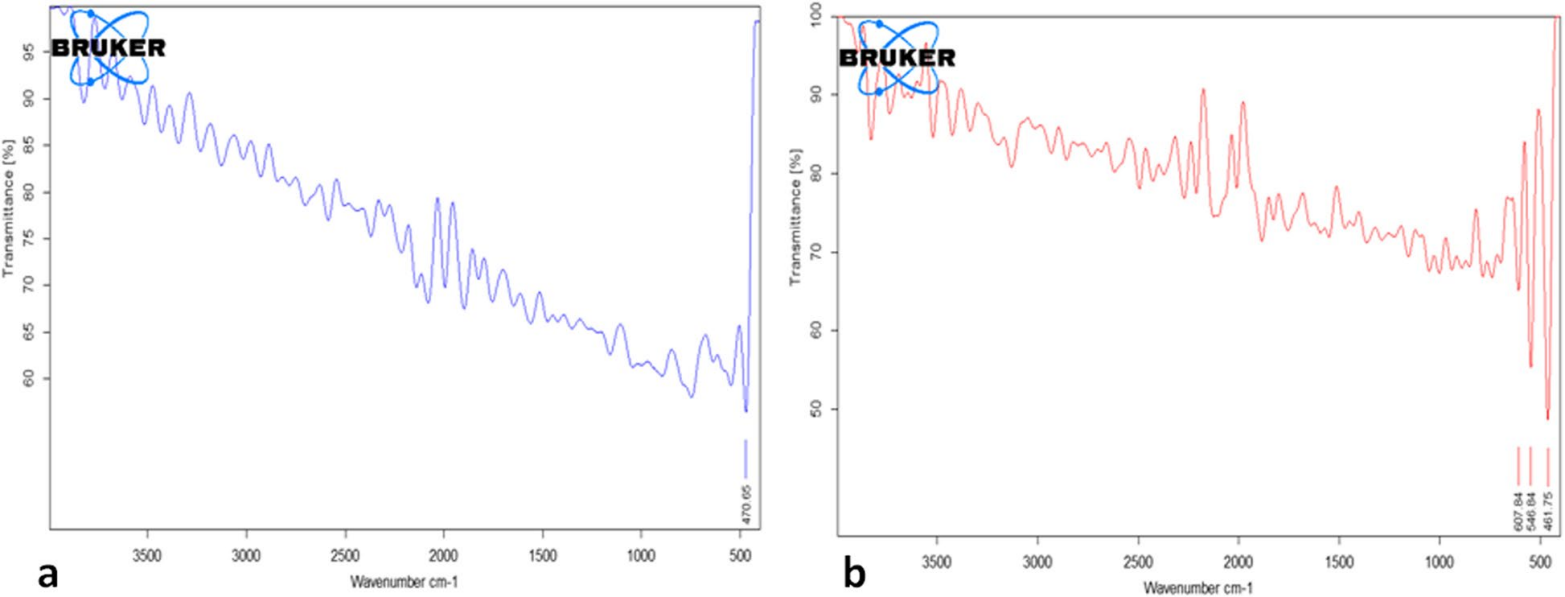
FTIR spectra of **a**) chitosan microsphere **b**) chitosan beads with the fungal pigment

**Fig. 9:**
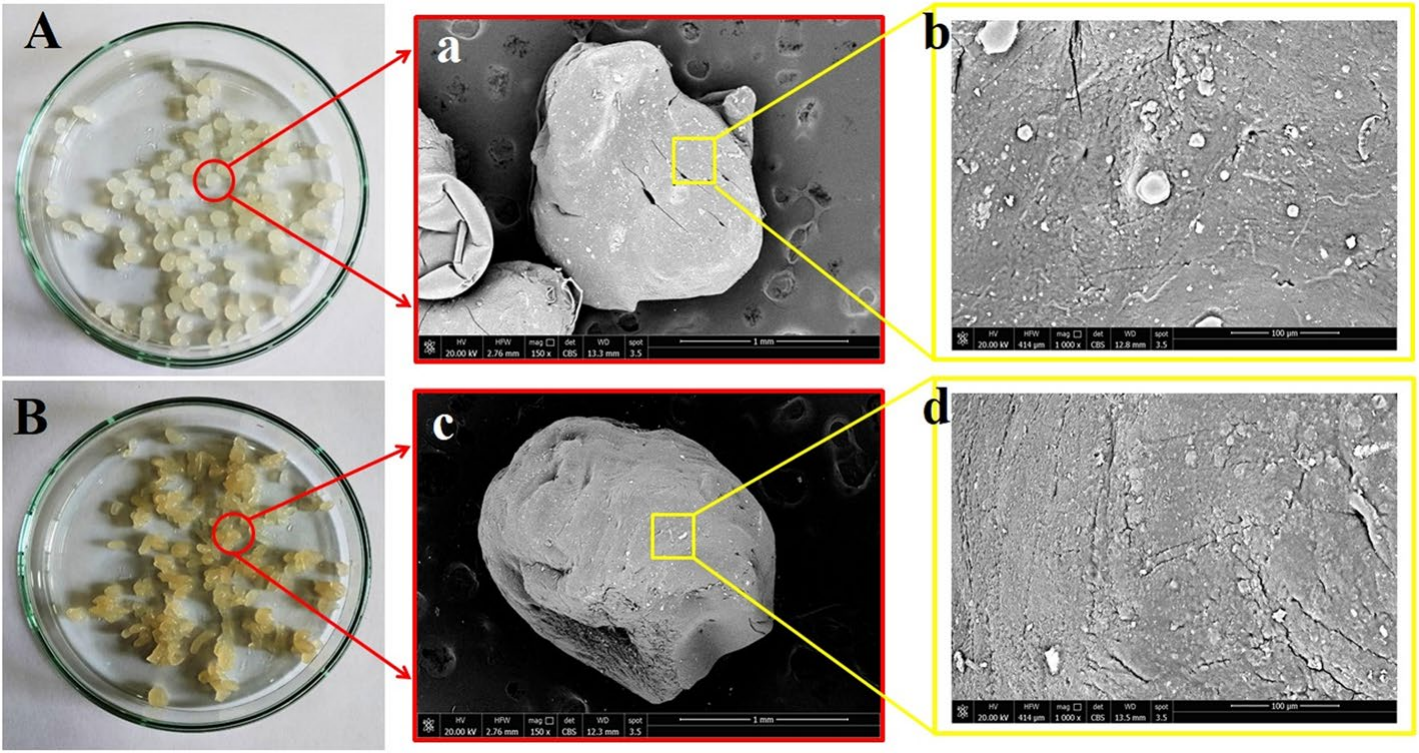
Visual observation of the prepared chitosan microspheres before (A) and after (B) pigment immobilization. a and b are scanning electron micrographs (SEM images) of the chitosan microspheres before pigment immobilization under low and high magnification power, respectively. While, c and d are SEM images of chitosan microspheres immobilized by the extracted *Fusarium* pigment under low and high magnification power, respectively

FTIR spectra of the free chitosan beads and chitosan beads loaded with the *Fusarium* pigment are presented in Fig. 8. While, scanning electronic microscopy images of both samples are represented in Fig. 9.

### Release experiment

**Fig. 10:**
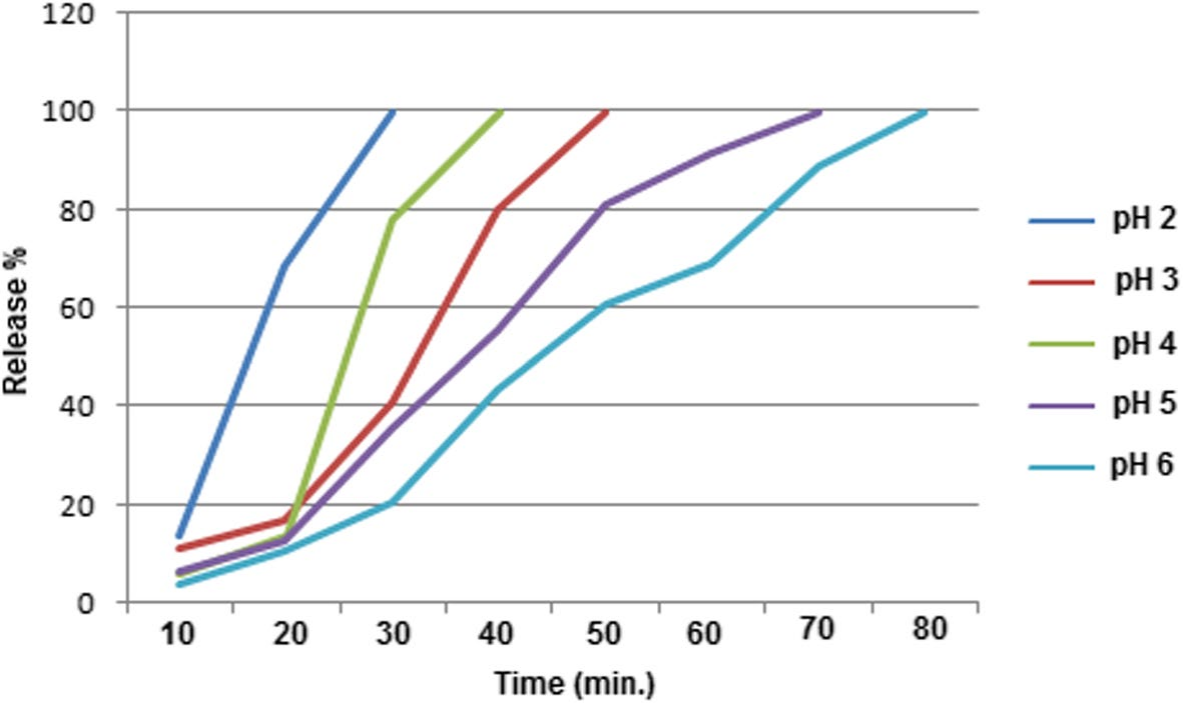
Release profiles of the *Fusarium* *verticillioides* pigment loaded in chitosan beads at pH 2.0, 3.0, 4.0, 5.0, and 6.0 at the room temperature

The pigment release from chitosan microspheres is represented in Fig. 10, which shows a plot of the percentage of pigment released as a function of time. As presented, the release was faster at pH 2.0, 3.0, and 4.0 than that at pH 5.0 and 6.0. Since, the pH influences how long it takes for pigment to permeate the medium from the microsphere's inner cavity. For a 50% pigment release, chitosan microspheres loaded with the fungal pigment took 17 (pH 2.0), 33 (pH 3.0) 25 (pH 4.0), 35 (pH 5.0) and 44 min (pH 6.0). Additionally, it was noted that within 30 min, the majority of the microspheres at pH 2.0 dissolved. It is evident that at pH6.0 the pigment was liberated from the chitosan within about 80 min.

## Discussion

Due to the high risks associated with most artificial pigments, scientists are becoming more and more interested in discovering natural sources of pigment [29]. In this work, *Fusarium verticillioides* AUMC 15934, a locally isolated and molecularly and morphologically identified fungal strain, was identified as a possible natural violet pigment producer. According to a report, *Fusarium verticillioides'* pigmentation determines its virulence in maize seedlings [30]**.** It was recently found that FvatfA controls the growth, stress tolerance, and production of mycotoxin and pigment in *Fusarium verticillioides* [31]. Here, the produced pigment was extracted and undergone yield estimation expressed by optical density (OD) using spectrophotometer. Similar methodology was used in a related study to extract red pigment from *Talaromyces purpurogenum* and evaluate its yield [14]. Furthermore, the pigment production of *Fusarium verticillioides* AUMC 15934 was optimized in a submerged culture by the one factor at a time approach for selecting the optimal medium and the response surface methodology approach for determining the ideal production process conditions. A related study described how the fungus *Fusarium verticillioides* optimized its ability to produce pigment [32]. While investigating the impact of different medium on biomass production and pigment yield, there was a converse correlation between the biomass quantity and the extracted pigment yield. Similar results were reported by [33]**.** Since, the overall biomass quantity was significantly less than the amount of extracellular pigments. Meanwhile, it was found that higher biomass quantity are significantly essential for fungal species that yield intracellular pigments [34].

When multiple factors influence a particular system, it is not feasible to screen and control for each factor's individual contribution as well as the impact of the interactions between different components. Economically and efficiently, this complexity can be reduced by using factorial designs. Response surface methodology (RSM) is a hypothetical statistical model-building technique used to illustrate a system that requires optimization [14, 35–37]. The obtained response (pigment yield) in the experimental zones was characterized in this study using the Box-Behnken’s design (BBD). As the determination coefficient (R^2^) gets closer to 1.0, a model becomes more accurate at predicting the response variable [37]**.** Consequently, the applied BBD model accurately described the pigment generation process, and the experimental data were appropriately fitted to the model at a significant level (P-value < 0.05). As a result, the applied BBD model adequately represented the pigment generation process, and the experimental data attained a significant (P-value < 0.05) fit to the model. In literature, the submerged fermentation process parameters, including fermentation time (0–336 h), agitation speed (100–200 rpm), temperature (21 °C–27 °C), and initial pH (4–9) were investigated and optimized for maximizing the production of orange and red pigments by BBD of RSM [38]**.** Using the statistical optimization method, it was discovered that the following settings would maximize the amount of pigment and biomass produced: 149 h as a fermentation time, 164 rpm as an agitation speed, 24 °C as a growth temperature, and an initial pH of 6.4 [38]**.**

By comparing the obtains in this study and the previous studies it was found that a reported *F. verticilloides* strain produced maximum naphthoquinone pigment by employing a basal growth medium with 20% (w/v) boiled potatoes, 2.5 g/l of yeast extract, 20 g/l of glucose, and, supplemented with 5 mg/l of KH_2_PO_4_, and adjusting the initial medium pH at 8.0 after 7 days incubation at 30 °C under agitation at 200 rpm [39]. While, the maximum yield of total naphthoquinones pigment from *Fusarium moniliforme* MTCC6985 was produced in potato dextrose broth (PDB) supplemented with 2%, yeast extract, glucose, and kH_2_PO_4_, adjusted at pH of 5.5, and incubated at 28 °C shaking incubator [40]. Another study isolated 26 isolates of *Fusarium* sp. from various agricultural fields and they showed different colored pigments. With optimized process parameters that included potato dextrose broth (2% w/v) supplemented by peptone (1% w/v) as a nitrogen source, glucose (2% w/v) as a carbon source, methionine (0.5%) as an amino acid and KH_2_PO_4_ (0.5%) as a metal salt under the incubation conditions of pH (5.5), temperature (28ºC), and incubation time (8 days) and metal salts, the pigment and biomass were produced at their highest productivity by the selected *Fusarium moniliforme* isolate [15]. While, the optimal conditions for red pigment formation in the submerged fermentation of *Fusarium solani* BRM054066, are agitation at 200 rpm and glucose concentration ≥ 20 g/L using Doehlert experimental model [41].

From literature, fungal pigments could be produced through different methods as follows: Pigment production through the submerged fermentation (Smf) was reported. Since, when *F. verticillioides* was cultivated in the modified potato dextrose broth, the maximum output of naphthoquinones was obtained [32]. Also, efficient pigment production was achieved efficient by *Fusarium moniliforme* after improvement of the composition of medium by submerged cultivation [15]. Solid state fermentation protocol was employed in the production of the red pigment from *Monascus purpureus* CMU001 using inexpensive crop residues as substrates (such as soybean meal, coconut residue, peanut meal, and corn meal). They found that supplementation of the corn meal by 8% (w/w) glucose resulted in the maximum pigment yield of 129.63 OD U/g of dry substrate [42]. Another study investigated the efficiency of using solid-state fermentation (SSF) using the brown rice powder to produce the red pigment (monascorubin) by *Penicillium minioluteum* ED24. A pigment yield of 4.89 mg/g of dry fermented substrate was produced under the optimum conditions including initial moisture content of 60%, 1% of soluble starch, 1% of peptone, KH_2_PO_4_ at 10% v/w, and trace ion solution at 10% v/w [43]. Pigment production through the fungal immobilization protocol was also reported. Since, pigment production was efficiently processed by *Penicillium purpurogenum* which was immobilized by 3% sodium alginate and the production was maximized by optimization of process parameters [44].

In the current study, spores of the tested fungal strain were exposed to 400 Gy of gamma radiation, which resulted in an increase in pigment production when compared to the respective control (non-irradiated). Gamma radiation's enhancement effect might result from its stimulatory effect on the production process, which involves causing mutations in the fungal cells' genomic DNA and the gene in charge of the production process [12]. Additionally, some fungi and bacteria produce more pigment and grow faster in response to ionizing radiation [45]. From an industrial point of view, there is a necessity to obtain a high yield from the produced strain. Thus, gamma irradiation mutagenesis helps in developing a mutant strains with a hyper-production that could be efficient for lowering the cost of the production process [23]**.** Microbial cells' physiology and bioactivity changed as a result of a series of events that happened after they were exposed to ionizing radiation. These changes can range from being transient to being permanent. These adjustments are based on the absorbed dose. Radiation stresses out cells more, which tends to mess with their layout. Because of this disruption in metabolism, irradiated cells display a range of changes [46]. Numerous research in the literature have proposed the use of gamma rays as a physical mutagen to improve microbial cultures and enhance their capacity for bioproduction and bioactivities. The enhancement role of gamma radiation (at 100 Gy) in the production of the natural pigment, Hypocrellin A by the *Shiraia* sp was reported [47]. The spore suspension of *Monascus* strains treated at a dose of 1 kGy produced the most modifications in the fungal morphology and pigment production [48]**.** Gamma irradiation of *Alternaria brassicae* at 750 Gy resulted in hyper production of the immunosuppressant Huperzine A [26]**.** Another study exposed some endophytic fungi to gamma radiation at the same dose range (200–800 Gy) as a sub-lethal doses and there was a modulation in the fungal tolerance potential against heavy metals [25]**.**

Chitosan was found to be a successful encapsulating agent for fungal pigment. Chitosan is frequently utilized as a cross linker in the formation of microspheres, in conjunction with glutaraldehyde. Covalent imine bonds are frequently formed between the amino groups of chitosan and aldehyde groups through this reaction. This results from a Schiff reaction that establishes resonance with nearby double ethylenic bonds. Covalent crosslinking creates a permanent network that improves the mechanical characteristics of the microspheres and permits free diffusion of water. Therefore, chitosan's mechanical characteristics and structure fall within a range that makes it appropriate for the controlled release of loaded agents [10].

## Conclusion

Many efforts have been made to increase pigment synthesis from natural sources as a way to scale up the manufacturing process to meet the needs of food applications. For microbial sources, these efforts have included the optimization of microbial cultivation techniques and strain mutagenesis induction. In this study, a new strain of *Fusarium verticillioides* AUMC 15934 was isolated for the production of a natural violet pigment. The pigment yield was maximized by optimizing the production media and process conditions as well as through the gamma irradiation employment at 400 Gy. To improve the natural pigment properties as maintaining its stability under environmental conditions and control their release under different pH degrees, the extracted pigment was successfully microencapsulated in a chitosan matrix cross-linked with sodium hydroxide and activated by glutaraldehyde. Thus, it is possible to view the encapsulated *Fusarium verticillioides ‘s* pigment in chitosan microspheres as a prospective natural violet colorant that could be added to food products.

## Data Availability

Data is provided within the manuscript.
